# Oxygen Consumption at 30 W of Exercise Is Surrogate for Peak Oxygen Consumption in Evaluation of Cardiorespiratory Fitness in Young-Adult African-American Females

**DOI:** 10.1155/2013/756276

**Published:** 2013

**Authors:** Richard M. Millis, Vernon Bond, M. Sadegh Asadi, Georges E. Haddad, Richard G. Adams

**Affiliations:** 1 Department of Physiology & Biophysics, The Howard University College of Medicine, Washington, DC 20059, USA; 2 Department of Health, Human Performance & Leisure Studies, The Howard University College of Medicine, Washington, DC 20059, USA; 3 Department of Neurology, The Howard University College of Medicine, Washington, DC 20059, USA; 4 Department of Medicine, The Howard University College of Medicine, Washington, DC 20059, USA

## Abstract

Body mass index (BMI) is negatively correlated with cardiorespiratory fitness, measured by maximal or peak oxygen consumption (VO_2peak_). VO_2peak_ measurements require heavy aerobic exercise to near exhaustion which increases the potential for adverse cardiovascular events. This study tests the hypothesis that VO_2_ measured at a fixed submaximal workload of 30 W is a surrogate for VO_2peak_. We studied 42 normotensive African-American female university students, 18–25 years of age. We measured VO_2peak_, blood pressure, and VO_2_ at a 30 W exercise workload and computed BMI. We found significant negative correlations between BMI and VO_2peak_ (*r* = −0.41, *P* < 0.01) and between BMI and VO_2_ at 30 W (*r* = −0.53, *P* < 0.001). Compared to VO_2peak_, VO_2_ at 30 W increased the significance of the negative correlation with BMI. The heart rate-systolic pressure product at 30 W was positively correlated with BMI (*r* = 0.36, *P* < 0.01) and negatively correlated with VO_2peak_ (*r* = −0.38, *P* < 0.001). The positive correlation between BMI and the heart rate-systolic pressure product and the greater negative correlation between VO_2_ and BMI at 30 W of exercise than that at exercise to fatigue suggest that normalized measurements of VO_2_ at the fixed exercise workload of 30 W could be useful surrogates for measurements of VO_2peak_.

## 1. Introduction

Aerobic exercise testing provides valuable data for measuring a person’s cardiorespiratory fitness and overall health. Such testing is also a basis for developing individualized, safe exercise prescriptions. Maximal and peak oxygen consumption (VO_2 max_, VO_2peak_) are gold standard measuring cardiorespiratory fitness [1]. However, low cardiorespiratory fitness makes it difficult for sedentary, overweight, and/or obese individuals to complete the high-intensity protocols required for VO_2 max_ or VO_2peak_ determinations [2]. Moreover, such exercise may put individuals with low cardiorespiratory fitness at risk for adverse cardiovascular events because determinations of VO_2 max_ and VO_2peak_ require substantial exertion to near exhaustion or fatigue [3, 4]. These limitations are consistent with the report that positive electrocardiographic indicators of cardiovascular disease are only 75% sensitive in women, compared to 90% sensitive in men and that African-American women appear to exhibit lower VO_2peak_ than a matched population of Caucasian women [3]. These findings suggest that lack of reliable measures of cardiorespiratory fitness at submaximal workloads may limit our ability to evaluate the health status and prescribe appropriate exercise regimens for women. These impediments have been addressed by the usage of submaximal aerobic exercise tests that are shown to be equally as reliable as VO_2 max_ and VO_2peak_ for measuring cardiorespiratory fitness in sedentary populations [4–9]. Although body mass index (BMI) is a reliable inverse correlate of VO_2 max_ and VO_2peak_ in most populations, no studies have been performed to determine the robustness of the correlation between BMI and VO_2peak_, compared to that at a fixed submaximal workload. Therefore, the present study tests the hypothesis that VO_2_ measured during submaximal exercise at a 30 W exercise workload is a surrogate for the VO_2peak_ measurement, as a correlate of BMI and, therefore, of cardiorespiratory fitness.

## 2. Methods

### 2.1. Subjects

Forty-two healthy young-adult women volunteered to participate in the study. Their anthropomorphic and physiological characteristics are summarized in Table 1. Participants were normotensive, free of any medication, nonsmoking, and nondrinkers. No participant engaged in regular physical activity and was informed of the study risks. The institutional review board at Howard University granted ethical approval, and informed consent was obtained from all subjects prior to study participation.

### 2.2. Study Protocol

Subjects participated in three separate sessions in the laboratory. The first session was used to familiarize the participant with the study monitors and devices. In the second laboratory visit, participants were instructed to abstain from exercise and caffeine or any energy drinks for 6 h and food for 3 h prior to entering the laboratory. Body height and weight were measured using standard laboratory procedures. The participant then performed a progressive test of VO_2peak_. Approximately 1–2 weeks after the second laboratory visit, participants performed the third laboratory visit. Prior to entering the laboratory, the participants were reminded of the prior physical activity and fasting instructions upon entering the laboratory. The participant then performed a submaximal steady-state exercise test using a work output of 30 W.

### 2.3. Peak Oxygen Consumption Test

VO_2peak_ was measured during a standardized incremental cycle task with a SensorMedics Ergoline-800 ergometer (SensorMedics Corp., Yorba Linda, CA). Participants were instructed to cycle continuously at 70–75 rpm, at a starting work intensity of 25 W. The work rate was increased by 25 W every 3 min until volitional fatigue. During the incremental exercise test, expired gas fractions of VO_2_, carbon dioxide, and minute ventilation (expired) were measured using the method of open-circuit indirect calorimetry (Physio-Dyne Max II Metabolic System, Quogue, NY). The gas analyzers were calibrated using known medical grade gas concentrations. The pneumatic gas volume was calibrated using a 3-L syringe. The VO_2_ value achieved during the last minute of the incremental exercise test was defined as VO_2peak_.

### 2.4. Submaximal Exercise Test

Participants cycled on the ergometer at an absolute work output of 30 W for a duration of 10 min. This low intensity work load of 30 W was selected because of the sedentary lifestyle of the study participants. Prior to the study, the electric brake ergometer was calibrated. Prior to the submaximal steady-state workload, the participants were instrumented with the SunTech Tango (SunTech Medical Inc., Raleigh, NC) automated blood pressure monitor that gates the R-wave with the Korotkoff sound to determine blood pressure. Heart rate was determined by electrocardiograph recordings of three electrodes positioned at the RA, LA, and V_5_ anatomical positions using the automated blood pressure device. Baseline blood pressure and heart rate measures were collected during the last 5 min of a 10 min sitting rest position. After baseline recordings, the participants performed 10 min of submaximal exercise on the cycle ergometer at a work intensity of 30 W. Heart rate, systolic and diastolic blood pressure were recorded during the last minute of the exercise.

### 2.5. Statistical Analysis

Pearson’s product-moment coefficient (*r*) and parametric linear regression analysis were used to compare the one-sided significance of correlations between BMI and VO_2peak_, between BMI and VO_2_ at 30 W of exercise, and between BMI and the heart rate-systolic blood pressure product at 30 W of exercise (Microsoft Excel, 2007).

## 3. Results

**Figure 1:**
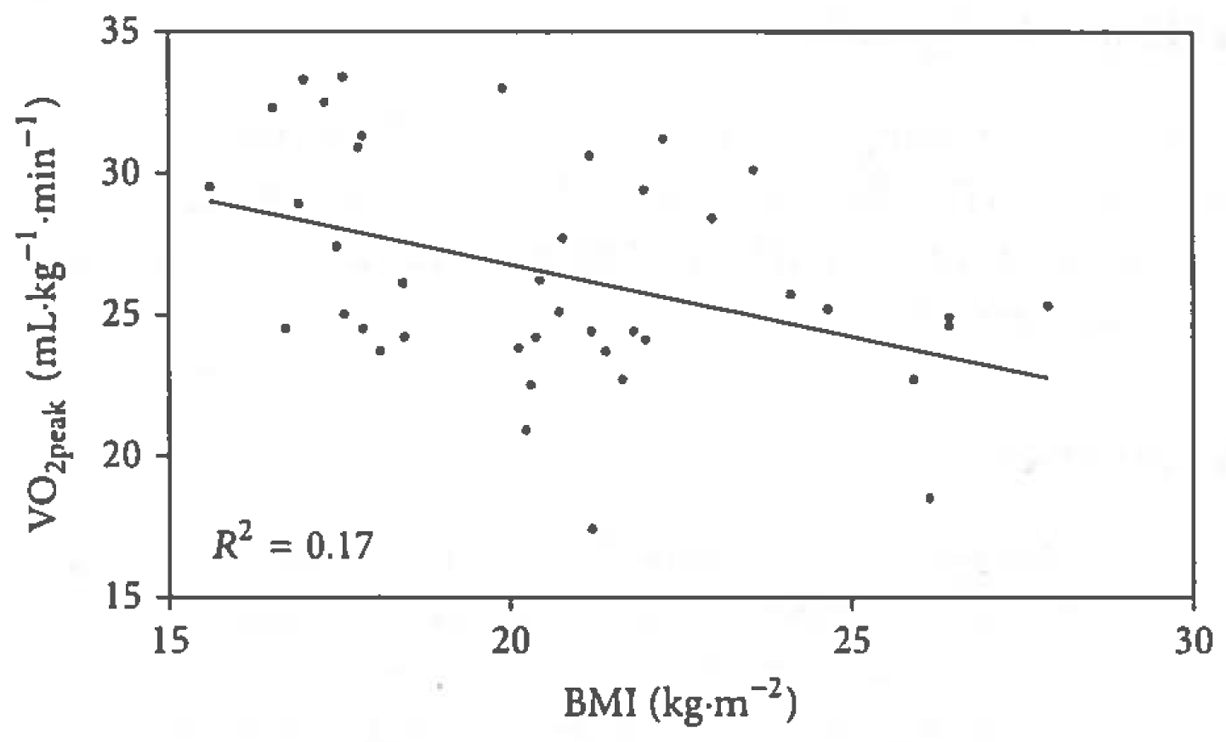
Linear regression analysis of the relationship between body mass index (BMI) and peak oxygen consumption (VO_2peak_). Subjects were 42 disease-free, normotensive, sedentary young-adult African-American females. VO_2peak_ was found to be significantly correlated with BMI (*r* = −0.41, *P* < 0.01).

**Figure 2:**
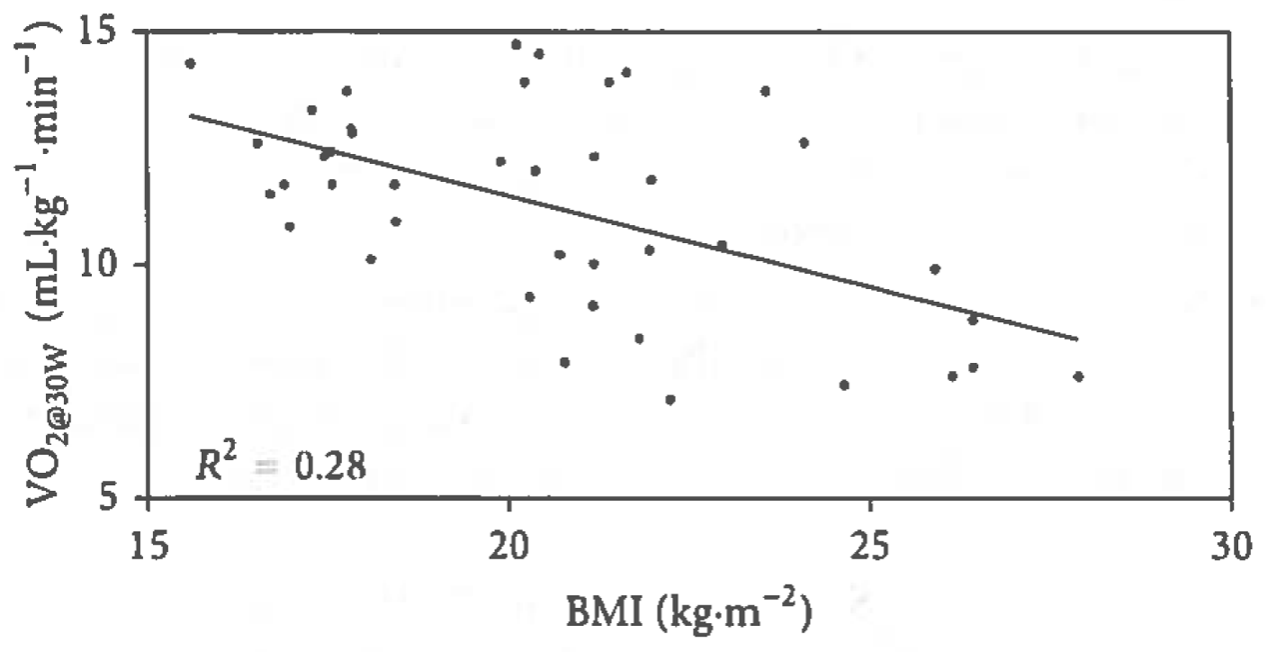
Linear regression analysis of the relationship between body mass index (BMI) and oxygen consumption (VO_2_) at the fixed submaximal workload of 30 W. Subjects were 42 disease-free, normotensive, sedentary young-adult African-American females. VO_2_ was found to be significantly correlated with BMI (*r* = −0.53, *P* < 0.001).

Table 1 presented the anthropomorphic and physiological characteristics of the study population. The subjects were mainly normotensive young-adult women with sedentary life style and hence low levels of cardiovascular fitness and low correlating VO_2peak_ levels. Figure 1 depicts the results of linear regression analysis demonstrating a significant negative correlation between both BMI and VO_2peak_ (*r* = −0.41, *P* < 0.01). The negative correlation between body mass and VO_2peak_ is not shown (*r* = −0.45, *P* < 0.001). Figure 2 presents the linear regression analysis and significant negative correlation between BMI and VO_2_ at 30 W of exercise (*r* = −0.53, *P* < 0.001). The negative correlation between body mass and VO_2_ at 30 W of exercise is also not shown (*r* = −0.55, *P* < 0.001). Correlations between BMI or body mass and heart rates and systolic and diastolic blood pressure at 30 W of exercise were not significant (*P* > 0.1). Both BMI and body weight were positively correlated with the heart rate-systolic pressure product at 30 W of exercise (*r* = 0.36, *P* < 0.01 and *r* = 0.39, *P* < 0.001, resp.). The heart rate-systolic pressure product was negatively correlated with VO_2peak_ (*r* = −0.38, *P* < 0.001).

## 4. Discussion

This study is the first to compare significance of the correlation between BMI and VO_2peak_ to that between BMI and VO_2_ at a fixed, submaximal exercise workload of 30 W in a disease-free population. The participants of this study were normotensive African-American female university students, 18–25 years of age, nonsmokers, nondrinkers, and free of any medication. The main finding of this study is a more significant correlation between BMI or body weight and VO_2_ at the fixed workload of 30 W than that between BMI or body weight and VO_2peak_. Overweight or obese subjects often experience difficulty and adverse cardiovascular events while performing cardiorespiratory fitness tests requiring maximal or fatiguing exertion. A similar study has not been performed in another population. Thus, the correlation coefficients reported herein cannot be compared to those reported in previous studies.

Heretofore, the popular wisdom was that VO_2 max_ or VO_2peak_ are the most reliable measures of aerobic capacity and, therefore, cardiorespiratory fitness [3, 4], However, it is reported that several submaximal exercise protocols such as perceptually regulated, graded exercise with computation of an aerobic power index, step tests, and dance tests provide reliable alternatives to VO_2 max_ or VO_2peak_ for measuring cardiorespiratory fitness [4–8]. Measuring cardiorespiratory fitness by submaximal exercise testing and estimating workload at a fixed heart rate are also a promising approach, yielding highly linear, significant correlation coefficients between heart rates and workloads >0.9 [10].

Measurements of cardiorespiratory fitness using exercise tests at submaximal workloads can help determine occupational fitness and evaluate work-related disabilities associated with jobs requiring large physical workloads [11]. Another important use of submaximal cardiorespiratory fitness tests is to evaluate the advertised safety, cost-benefit, and health outcome claims of exercise and dietary regimens [12]. The association of low VO_2peak_ and decreased motor strength in a population of 60-year-old healthy men [11] suggests that submaximal cardiorespiratory fitness testing, physical therapy counseling, and interventions in such populations might decrease their high rate of daily activity- and work-related injuries, as well as the associated health care costs. The further importance of screening such a population for cardiorespiratory fitness is underscored by a report that low cardiorespiratory fitness is associated with high risk for sudden cardiac death in a population of middle-aged men [13]. Thus, results of the present study imply that cardiorespiratory fitness can be reliably measured in populations of overweight, elderly, or otherwise frail subjects by cycle ergometer exercise at a workload of 30 W, thereby reducing the potential for adverse cardiovascular events.

This is also the first study to show a significant negative correlation between VO_2peak_ and the heart rate-systolic pressure product, as well as positive correlations between BMI or body weight and the heart rate-pressure product during aerobic exercise at a fixed submaximal workload. The finding of significant correlation between body mass and the heart rate-pressure product, a measure of myocardial oxygen demand [14], indicates a significant association of an increased requirement for coronary blood flow during exercise in overweight or obese compared to normal-weight persons. The coronary is the circulation with the lowest venous oxygen content, oxygen extraction ratio, and, therefore, oxygen demand at rest. Increases in myocardial oxygen demand must be met, mainly, by increases in coronary blood flow which, when compromised, can result in adverse cardiac events and sudden death [15]. The fact that coronary blood flow is limited by arterial narrowing in atherosclerosis is well known [16], but other causes such as smoking, nicotine, and cocaine use are less well appreciated. Coronary arterial luminal diameters and areas are shown to be significantly smaller in females than in males, as well as in overweight than in normal-weight individuals [17]. Normotensive African-Americans, especially women, have also been shown to have limitations of endothelial function known to affect the coronary circulation [18, 19]. Therefore, our finding of a significant association between large body mass and large heart rate-pressure product during exercise at 30 W in disease-free normotensive, sedentary young-adult females may be indicative of the potential for limitations of coronary blood flow linked to adverse cardiac events associated with aerobic exercise, smoking, and cocaine use in this population [20]. This finding also supports the hypothesis that experiencing adverse cardiac events during exercise could explain lack of participation of persons with low aerobic capacity in exercise programs [21], thereby creating a vicious cycle of exercise avoidance, omitting effective strategies for weight loss, and improving cardiorespiratory fitness.

## 5. Limitations and Conclusions

Limitations of this study were (1) inclusion of only sedentary subjects with a relatively low level of cardiorespiratory fitness, thereby limiting our ability to extrapolate the results to a wider range of aerobic capacity; (2) exclusion of obese subjects; (3) not randomizing the exercise procedures and, therefore, not varying the order of presentation and measurement of the VO_2peak_ test before and after measuring VO_2peak_ at the submaximal, 30 W workload; and (4) performing the cardiovascular measurements such as heart rate and blood pressure only during the submaximal exercise trial. These cardiovascular measurements were made to determine whether the study subjects exhibited physiological responses at this submaximal, absolute workload and whether the subjects exhibiting the highest cardiorespiratory fitness during the VO_2peak_ test would exhibit the lowest cardiac oxygen demand at the submaximal 30 W workload, as expected.

In summary, this study demonstrates significant associations between large body mass, low oxygen consumption, and high myocardial oxygen demand during aerobic exercise at a fixed workload of 30 W in a population of normotensive, sedentary, young-adult African-American females. The greater correlations between BMI or body weight and oxygen consumption found at 30 W of submaximal exercise than those between BMI or body weight and VO_2peak_ in this population suggest that normalized measurements of VO_2_ during exercise at submaximal workloads may be useful surrogates for measurements of VO_2peak_ to limit adverse cardiac events without loss of reliability in evaluations of cardiorespiratory fitness.

## Figures and Tables

**Table 1:** Descriptive characteristics of the study subjects.

Variables	Subjects (*n* = 42)
Age (yr)	20.7 ± 2.2
Height (cm)	165.3 ± 7.9
Weight (kg)	68.4 ± 11.7
VO_2peak_ (mL·kg^−1^·min^−1^)	26.4 ± 3.9
HR_peak_ (beats·min^−1^)	182.3 ± 12.3
VO_2@30 W_ (mL·kg^−1^·min^−1^)	11.2 ± 2.2
HR_@30 W_ (beats·min^−1^)	80.9 ± 12.3
Systolic pressure at 30 W (mm Hg)	119.0 ± 2.8
Diastolic pressure at 30 W (mm Hg)	76.8 ± 2.2
Rate-pressure product at 30 W (bpm·mm Hg)	9,445 ± 1,589

HR_peak_: peak heart rate; VO_beak_: peak oxygen consumption. HR_@30 W_: heart rate during exercise at the submaximal workload of 30 W; VO_2@30 W_: oxygen consumption during exercise at the submaximal workload of aerobic exercise. Systolic pressure, diastolic pressure, and rate-pressure product at 30 W = systolic blood pressure, diastolic blood pressure, and heart rate × systolic pressure product during exercise at the fixed workload of 30 W. Data are means ± standard deviations.
